# Prenatal Exposure to Tobacco and Cannabis in Six Race/Ethnicity Groups during the First Three Years after Legalization of Cannabis for Recreational Use in California

**DOI:** 10.3390/ijerph21010011

**Published:** 2023-12-21

**Authors:** Martin Kharrazi, Kimberly Berger, Michelle Pearl, Ying Li, Josephine DeGuzman, Paramjit Behniwal, Allison Morse, Ilya Moskalenko, Rebecca J. Williams, Jianwen She

**Affiliations:** 1 Sequoia Foundation, Berkeley, CA 94710, USAilya@sequoiafoundation.org (I.M.); 2 Environmental Investigations Branch, California Department of Public Health, Richmond, CA 94804, USA; 3 Environmental Health Laboratory Branch, California Department of Public Health, Richmond, CA 94804, USAparamjit.behniwal@cdph.ca.gov (P.B.);; 4 Genetic Disease Screening Program, California Department of Public Health, Richmond, CA 94804, USA; 5 California Tobacco Prevention Program, California Department of Public Health, Sacramento, CA 95814, USA; rebecca.williams@cdph.ca.gov

**Keywords:** tobacco, cannabis, pregnancy, cannabis legalization, cotinine, THC, biomarker, 11-hydroxy-Δ9-tetrahydrocannabinol

## Abstract

There are known health concerns linked to prenatal tobacco and cannabis exposures. This study aims to objectively determine the level of exposure to tobacco and cannabis in pregnant individuals from six race/ethnicity groups (Black, Hispanic, Asian Indian, Native American, Vietnamese, and White) in the first three years following legalization of recreational marijuana use in 2018 in California. We used a cross-sectional sample of prenatal screening program participants (2018–2020) from southern and central California (N = 925). Exposures were estimated by a lab analysis of cotinine (tobacco) and 11-hydroxy-Δ9-tetrahydrocannabinol (OH-THC, cannabis) in banked serum. Disparities in tobacco exposure were evident, with Black subjects experiencing the highest smoking rate (16%) followed by Native American (10%) and White (8%) subjects, and ≤2% among Hispanic, Asian Indian, and Vietnamese subjects. Environmental tobacco exposure generally showed a similar pattern of exposure to tobacco smoking across race/ethnicity groups. Cannabis detection ranged from 5% among Hispanic subjects to 12% and 13% among White and Black subjects, respectively, and was higher among tobacco users and those exposed to environmental tobacco smoke than those with no cotinine detected. Tobacco and cannabis exposure were generally greatest in younger subjects and those with indices of a lower economic status; however, among Black subjects, cannabis exposure was greatest in older subjects and those with a higher socioeconomic status. Race/ethnicity, age, and socioeconomic factors can inform targeting of high-exposure groups for intervention.

## 1. Introduction

Prenatal tobacco exposure through active smoking or environmental tobacco smoke (ETS) is one of the most significant avoidable causes of maternal and child morbidity and mortality [1]. Effects of prenatal smoking manifest in utero with slowed fetal growth and preterm birth and in childhood with an increased risk of adverse respiratory, neurological, and cardiometabolic effects [2,3], and may extend to subsequent generations with increased asthma and body mass [4,5]. Tobacco smoke contains thousands of chemicals including nicotine, carbon monoxide, heavy metals, polycyclic aromatic hydrocarbons, and others known to harm human health [1]. The health effects of prenatal ETS exposure are similar and consistent, although not as strong as for active smoking [1,3,6,7].

Cannabis use during pregnancy can also harm fetal and long-term development [8]. Babies exposed to cannabis prenatally are more likely to have restricted growth in utero, lower birth weight, and smaller head circumference [9,10]. In addition, emotional processing, sleep, aggressive behavior, and the ability to pay attention may be affected in infants [11,12]. Prenatal cannabis exposure also lowers immune response to viruses and causes ventricular septal defects [13,14]. Children exposed to cannabis in utero are at a greater risk for delinquent behavior, lower IQ, decreased attention span, and using cannabis and nicotine products themselves [9,10]. While many of the same toxic chemicals in tobacco smoke such as heavy metals, carbon monoxide, benzene, and formaldehyde are found in cannabis smoke, some are unique cannabinoid compounds found specifically in cannabis smoke such as tetrahydrocannabinol (THC), the psychoactive chemical in cannabis [15]. The four major compounds in cannabis are delta-9-tetrahydrocannabinol (Δ9-THC), cannabidiol (CBD), delta-8-tetrahydrocannabinol, and cannabinol (CBN) [16,17]. As of 1 January 2018, the adult use of cannabis was legal under California law (Medicinal and Adult-Use Cannabis Regulation and Safety Act) [18]. After State review, cannabis smoke and Δ9-THC were included in the Proposition 65 warning due to developmental toxicity in 2020 [19].

Over the past several years, there have been declines in cigarette smoking during pregnancy in the United States, from 7.2% in 2016 to 4.6% in 2021 [20]. This trend was seen across age and race groups, with the largest declines among mothers under age 30 and non-Hispanic Asian mothers [21]. While cigarette smoking has declined, other sources of nicotine such as e-cigarettes have emerged over time, including the use of multiple products [22]. About 1.3% of pregnant women nationally used e-cigarettes during the last 3 months of pregnancy [23]. People report the belief that e-cigarettes are less toxic, conspicuous, and harmful to passively exposed individuals than cigarettes [24]. Meanwhile, the percentage of pregnant women reporting cannabis use has increased from 3.4% in 2002–2003 to 7.0% in 2016–2017 [25]. The prevalence of cannabis and tobacco co-use among adults is 14.0% [26], with co-use among pregnant women as high as 84.5% of those reporting tobacco use during pregnancy [27]. These trends are in line with cannabis usage among women generally, which occurs at higher rates than in the pregnant population [28,29]. The main reported reasons for using cannabis during pregnancy are stress, anxiety, nausea, and pain [21].

Disparities in cigarette and cannabis use during pregnancy exist across demographic characteristics. For example, Non-Hispanic American Indian/Alaska Native women are more likely to smoke during pregnancy (16.8%) than non-Hispanic White women (10.5%), Non-Hispanic Black women (6.0%), and Hispanic women (1.8%) [30]. Further, smoking during pregnancy is highest for women with a high school diploma or GED (12.2%) and lowest among women with some college or an associate degree (7.9%). Similarly, rates of cannabis use during pregnancy are higher among American Indian/Alaska Native women (48%) compared to rates among White, Black, and Hispanic participants (ranging from 28 to 35%) and rates are lower among women with at least some college education (30%) compared to those with a high school diploma or less (43%) [31].

In response to the harm that tobacco and cannabis pose to an unborn child and mother, public health and medical practitioners have instituted interventions and media campaigns [32] to educate women about smoking and secondhand exposure during and after pregnancy [33,34]. Many programs, including counseling and mobile health programs, exist to help smokers to quit [35,36]. The American College of Obstetrics and Gynecology has also made recommendations that pregnant women avoid cannabis [37]. Despite these programs and recommendations, only ~20% of tobacco smokers report quitting in the 3 months before pregnancy, and another 20% report quitting early in pregnancy [38].

A common method of ascertaining exposure to cannabis and tobacco products is self-reporting. Exposure rates based on biomarkers are higher than those based on self-report [39]. Selection bias may also exist in these studies, especially given the sensitivity around using these products. Participant selection and self-reporting can be differentially biased across race/ethnicity groups [40]. Little biomarker-based data on tobacco and cannabis exposure are available for certain racial/ethnic populations, such as Native American and Asian subgroups. Knowledge about tobacco and cannabis exposure obtained using unbiased methods is important to more accurately identify disparities and help target behavioral and other types of interventions toward the most highly exposed groups.

This paper aims to (i) objectively determine the level of exposure to tobacco and cannabis in pregnant women from six race/ethnicity groups (Black, Hispanic, Asian Indian, Native American, Vietnamese, and White) in the first three years following legalization of recreational marijuana use in 2018 in California, (ii) find demographic, socioeconomic, and neighborhood factors that identify subgroups with the highest tobacco and cannabis exposures within race/ethnicity groups, and (iii) assess whether prenatal exposure to tobacco is associated with exposure to cannabis in six race/ethnicity groups. These six race/ethnicity groups were included in a previous tobacco exposure study in California and are expected to have a range of tobacco and cannabis exposures [41].

## 2. Materials and Methods

**Figure 1:**
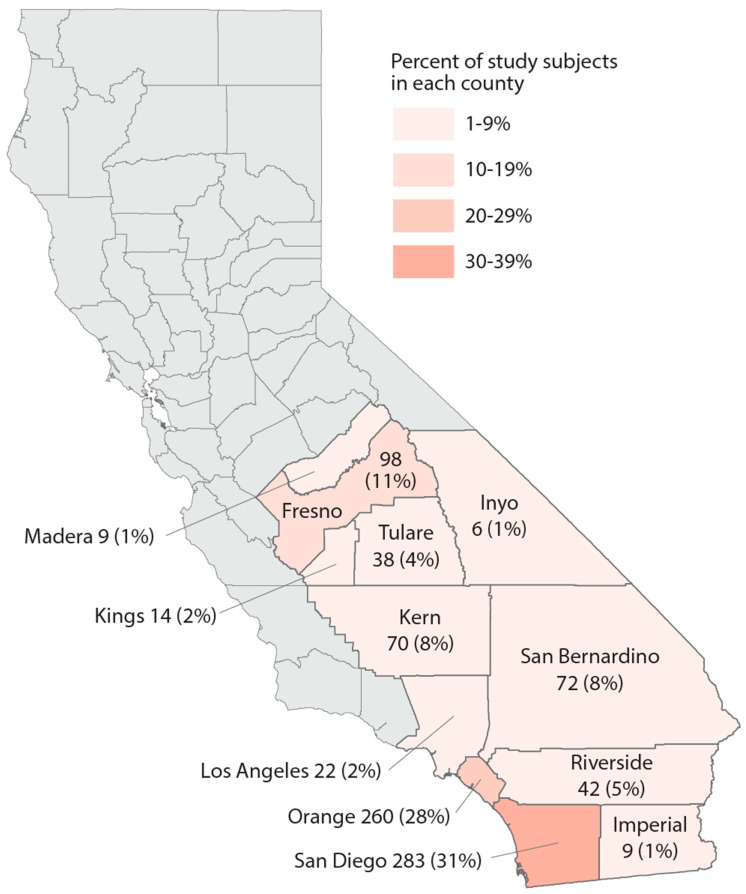
Map of the study region, number, and percent of study subjects in 13 California counties, sample of 925 California prenatal screening program enrollees, 2018–2020.

Study population. We utilized data from the California Biobank Program to randomly select subjects who participated in the California Prenatal Screening Program in 2018–2020 and who reported their race/ethnicity as Black, Hispanic, Asian Indian, Native American, Vietnamese, or White. Following prenatal testing at two prenatal screening laboratories, specimens from second trimester screening (15–20 weeks of gestation) were stored, unless screening participants opted out of storage (estimated at 6.5%) [42]. The study population included residents from 12 counties as well as some from the bordering area of Los Angeles County (Figure 1). A random sample of 100 participants per year was selected within each of the 6 race/ethnicity groups, for a total of 1800 subjects. Serum specimens were stored at −70 degrees Celsius prior to retrieval from the California Biobank Program freezers in August 2021, at which point specimens were randomized into batches of ~160 for lab processing.

Laboratory methods. A combined tobacco/cannabis serum assay using high-throughput 96-well plate positive pressure and hybrid solid phase extraction (HT-96-PP-Hb-SPE) liquid chromatography–tandem mass spectrometry was developed for this study. The method used formic acid, citric acid, and sonication (pre-SPE step) to break up cotinine- and tetrahydrocannabinol-binding proteins in 100 μL of serum. Sample clean-up was then achieved via 96-PP-Hb-SPE. The analysis was conducted with an Agilent 1290 Ultra High Performance Liquid Chromatography System coupled with an Agilent 6460 Triple Quadrupole Mass Spectrometer (UHPLC-ESI-MS/MS) (Agilent Technologies, Inc., Santa Clara, CA, 95051 USA) in positive electrospray ionization and multiple reaction monitoring (MRM) mode. Cotinine was analyzed as a biomarker for tobacco exposure, and 11-hydroxy-delta-9-tetrahydrocannabinol (OH-THC), Δ9-THC, and CBN were analyzed as cannabis exposure biomarkers. Isotope-labeled internal standards (cotinine-d3, Δ9-THC-d9, OH-THC-d3, and CBN-d3) were employed to compensate for analyte losses and potential matrix effects and instrument response variations. Due to analytical issues with quantifying Δ9-THC and CBN, results for these analytes are not reportable; therefore, OH-THC was used to define cannabis exposure in this paper. Due to time constraints, only 931 serum specimens were analyzed out of the 1800 originally selected. Specimens were randomly selected for the analysis, and there was no significant difference between the specimens that were and were not analyzed with respect to age and socioeconomic characteristics. In addition, results from some specimens did not meet laboratory result acceptability criteria (254 for OH-THC and 6 for cotinine), resulting in 925 subjects with reportable cotinine and 677 with reportable OH-THC.

Study variables. Data provided by the prenatal screening program were used to geolocate residences (latitude and longitude coordinates at the second decimal level), and to obtain information on subject race/ethnicity, age at time of blood collection, and Medicaid insurance coverage. Subjects self-reported race/ethnicity on the program’s test request form. The hierarchy used to assign race/ethnicity in the case of multiple selections was as follows: 1. Native American, 2. Vietnamese, 3. Asian Indian, 4. Hispanic, 5. Non-Hispanic Black, and 6. Non-Hispanic White. This order was selected to maximize subject numbers. Neighborhood level data on poverty and unemployment were obtained from the 2016–2020 American Community Survey based on the census tract of residence. The California Bureau of Cannabis Control (CBCC) 2018–2020 licensure files were used to obtain cannabis retailer addresses and to estimate the date ranges of operation. Missing address data were located via manual scraping of websites such as OpenCorporates, Google Maps, and Yellow Pages using the corporation names gathered from the CBCC. RStudio (Posit Corp., Boston, MA, USA) was used to estimate the haversine distance between each study subject’s residence and each cannabis retailer in California.

Data analysis. The shape of the serum cotinine distribution was bimodal with the small right mode representing exposure levels equivalent to smoking (hereafter, “smokers”), and a large log-normal mode representing nonsmokers with exposure to ETS. In the six race/ethnicity groups, 3–7% of subjects had a cotinine value of 0. We used the cotinine thresholds recommended by Benowitz et al. [43] to most accurately identify smokers. For adults (age > 19 years), the cutoffs were ≥5 ng/mL for non-Hispanic White subjects, ≥6 ng/mL for non-Hispanic Black subjects, and ≥1 ng/mL for Mexican Americans (applied to all Hispanic subjects due to lack of data on country of origin). For adolescents (age 12–19 years), the cutoffs were ≥3 ng/mL for non-Hispanic White and Black subjects, and ≥1 ng/mL for Mexican American (Hispanic) subjects. A smoker cutoff of ≥3 ng/mL was used for our Asian Indian, Native American, and Vietnamese groups. We calculated smoking rates (%) in each race/ethnicity group for purposes of comparison. These smoking rates can be interpreted as the percentage of subjects with the highest exposure to tobacco, regardless of the source of nicotine. Due to small numbers of smokers, we did not analyze the level of cotinine among smokers continuously.

Study subjects with serum cotinine values below smoker thresholds were designated as nonsmokers in their respective age/race/ethnic groups. In categorical analyses, subjects exposed to ETS were defined as those with cotinine ≥0.1 ng/mL (the detection limit) but below the race/ethnicity-specific cutoff points for smokers. Lack of exposure to ETS was defined as having a cotinine value < 0.1 ng/mL (i.e., not detected by the assay). In continuous analyses of ETS in nonsmokers, we used log_10_-transformed cotinine values above and below the method detection limit (0.1 ng/mL) for subjects with values > 0 ng of cotinine/mL of serum. Values below the detection limit were included in the analysis, rather than assigning a constant such as 0.1/√2 ng/mL, to more precisely estimate the log-normal distribution of ETS exposure in study subjects (Supplemental Figure S1). Across race/ethnicity groups, 30–55% of cotinine values were below the detection limit. To compare ETS exposure levels (concentrations) across race/ethnicity groups, the geometric mean of cotinine values (with 95% confidence intervals) and interquartile range in nonsmokers were calculated.

The analysis of cannabis exposure was limited to the subgroup of the study population with OH-THC values (N = 677 subjects). In categorical analyses, subjects with ≥1.0 ng of OH-THC/mL of serum (the method detection limit) were defined as cannabis-exposed and those with <1.0 ng of OH-THC/mL of serum were not exposed to cannabis in all race/ethnicity groups. These categories were used to calculate the percent of subjects with exposure to cannabis. We observed a bimodal distribution of OH-THC in each race/ethnicity group (Supplemental Figure S2): a lower mode with a zero value for OH-THC (40–90% of subjects) and a second log-normal mode. Log_10_-transformed OH-THC values were used for a continuous analysis of the exposure level among subjects with OH-THC values > 0 ng/mL. Values below the detection limit were included in the analysis, rather than assigning a constant such as 1/√2 ng/mL, to more precisely estimate the log-normal distribution of OH-THC. To compare OH-THC exposure across race/ethnicity groups, the geometric mean (with 95% confidence intervals) of OH-THC in subjects with values > 0 ng/mL was calculated along with the interquartile range. These means can be interpreted as the level (concentration) of exposure to cannabis in the second mode.

To identify the underlying high-exposure subgroups within six race/ethnicity groups for the five study exposure measures—tobacco smoking (%), ETS (% and level), and cannabis (% and level)—we conducted a bivariate exploration of the following factors: age at blood collection (<21, 21–27, 28–34, ≥35 years), insurance (subjects with Medicaid coverage, yes vs. no), poverty (subjects residing in census tracts with ≥20% of persons with household income below poverty level, yes vs. no), and unemployment (subjects residing in census tracts with ≥6% of persons in the labor force unemployed, yes vs. no). For tobacco smoking, ETS exposure, and cannabis detection, a subgroup was considered a “high-exposure subgroup” if prevalence was >5% higher than the overall race/ethnicity prevalence. For mean cotinine in nonsmokers and mean OH-THC, a subject was considered to be in a “high-exposure subgroup” if mean cotinine was 0.55 ng of cotinine/mL of serum or higher, or OH-THC was 0.096 ng/mL or higher than the race/ethnicity mean (equivalent to the smallest mean observed in all categories in all six race/ethnicity groups for that study exposure measure). Due to the exploratory nature of the subgroup analyses, no statistical comparisons were conducted. Subgroup analyses were conducted only for the 2–3 race/ethnicity groups with the highest rates or levels of the five study exposure measures because public health targeting would be most impactful in these groups.

Approval for the research project was obtained from the California Health and Human Services Agency Committee for the Protection of Human Subjects in 2019 (Project # 2019-049) and from the California Biobank Program in 2018 (SIS Request #1409).

## 3. Results

Study population description. Demographic and neighborhood-level attributes of subjects in each race/ethnicity group can be found in Table 1. The majority of study subjects resided in San Diego and Orange Counties, both urban centers (Figure 1). Across race/ethnicity groups, Black, Hispanic, and Native American subjects were younger and had the highest proportion covered by Medicaid or residing in impoverished areas. Asian Indian and Vietnamese groups had the oldest subjects and were most likely to reside in areas with less poverty and unemployment. Vietnamese subjects mainly resided in Orange County. In each race/ethnicity group, 21–28% of subjects lived within 3 km of a cannabis retailer; Vietnamese subjects experienced the highest density of cannabis retailers compared to other race/ethnicity groups. White subjects were generally intermediary with regard to these characteristics.

Tobacco and cannabis exposure. The findings for tobacco exposure measures for race/ethnicity groups are presented in Table 2a. The prevalence of detected cotinine ranged from 45% to 70% across the race/ethnicity groups. The highest prevalence of smoking was observed among Black, Native American, and White subjects (range: 8–16%); smoking prevalence among the other groups was ≤2%. A large percentage of each race/ethnicity group was exposed to ETS (range: 44–53%). Following the pattern observed for smoking, Black subjects had the highest percentage of nonsmokers exposed to ETS and the highest level (geometric mean) of cotinine, followed by Native American nonsmokers. Race/ethnicity groups generally ranked the same across tobacco exposure metrics, although Asian Indian nonsmokers had the third highest percentage exposed to ETS. Vietnamese nonsmokers had the lowest percentage of ETS exposure and one of the lowest levels across race/ethnicity groups. An evaluation of smoking rates and percent of ETS exposure and geometric means in nonsmokers over time (2018–2020) did not reveal any discernible time trends in any race/ethnicity group (data not shown).

The findings for measures of exposure to cannabis are presented in Table 2b. Quantifiable values of OH-THC (≥1.0 ng/mL) were found in all groups (range: 5–13%). Race/ethnicity group patterns of exposure to cannabis differed from those observed for tobacco exposures. Black and White subjects had the highest percentages and levels of cannabis exposure, and Asian Indian subjects had the third highest percentage of cannabis exposure (7.7%) and the fourth highest level. Hispanic subjects had the lowest percentage of cannabis exposure, but the third highest level. An evaluation of quantifiable OH-THC rates and geometric means over time (2018–2020) did not reveal any discernible time trends in any race/ethnicity group (data not shown).

The relation between tobacco exposure and cannabis exposure is presented in Table 2c. In categorical analyses, both tobacco smoking and ETS-exposed subjects had elevated rates of cannabis exposure in nearly all race/ethnicity groups. The range of quantifiable OH-THC across race/ethnicity groups was 8–50% among smokers, 7–14% among subjects with exposure to ETS, and 2–7% among subjects with nondetectable cotinine. Conversely, subjects exposed to cannabis had higher rates of exposure to tobacco in all race/ethnicity groups. The range of cotinine detected was 58–78% among subjects with quantifiable OH-THC and 41–70% among those with OH-THC not detected. A moderate positive correlation between cotinine and OH-THC was found in Black, Vietnamese, and White groups (range: 0.25–0.42 for subjects with non-zero values for both analytes).

**Figure 2:**
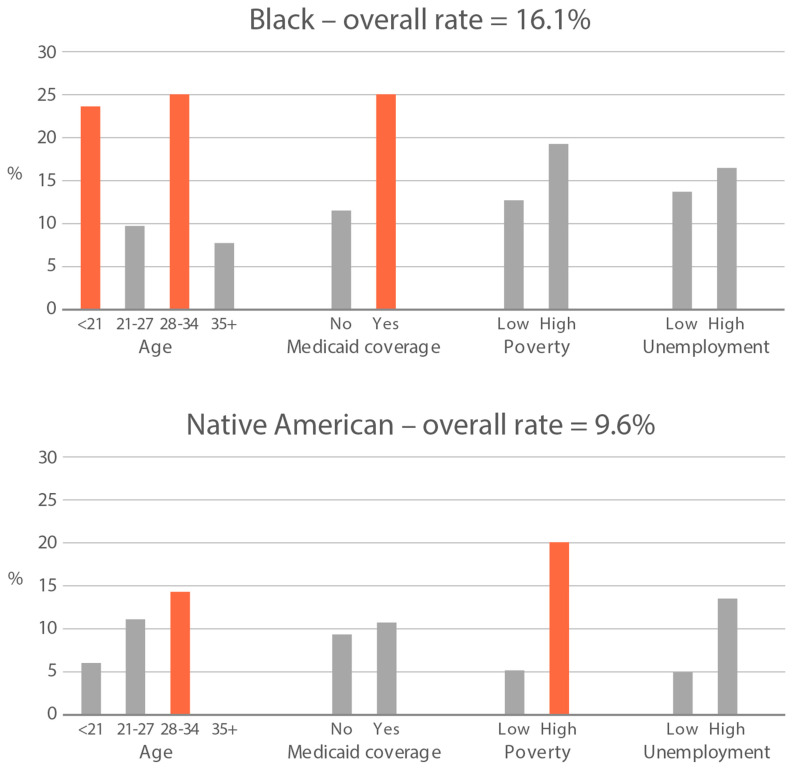

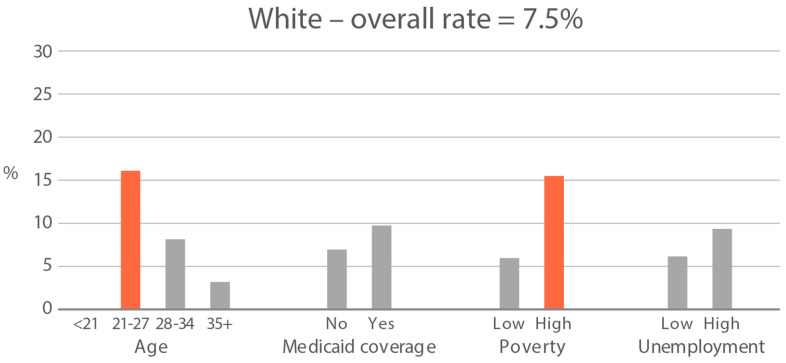
Smoking rate is defined by cotinine, as per Benowitz et al., 2008 [43] in age and socioeconomic subgroups: Black, Native American, and White high smoking race/ethnicity groups. Highlighted bars indicate high-exposure subgroups.

**Figure 3:**
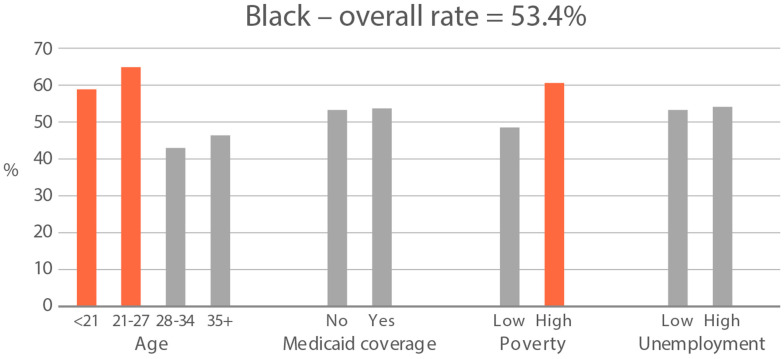

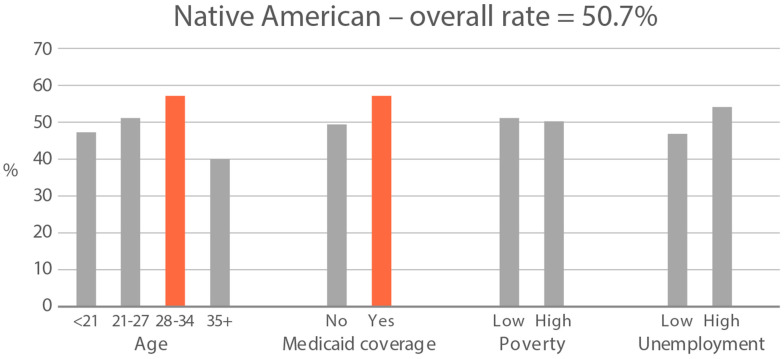
Percent of nonsmokers with cotinine detected in age and socioeconomic subgroups: Black and Native American high-ETS-exposure race/ethnicity groups. Highlighted bars indicate high-exposure subgroups.

**Figure 4:**
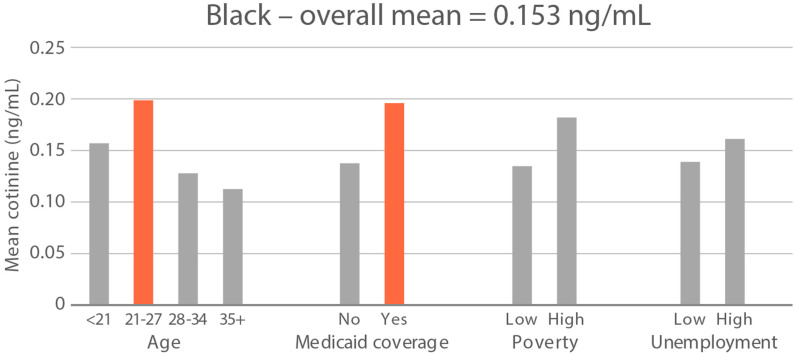

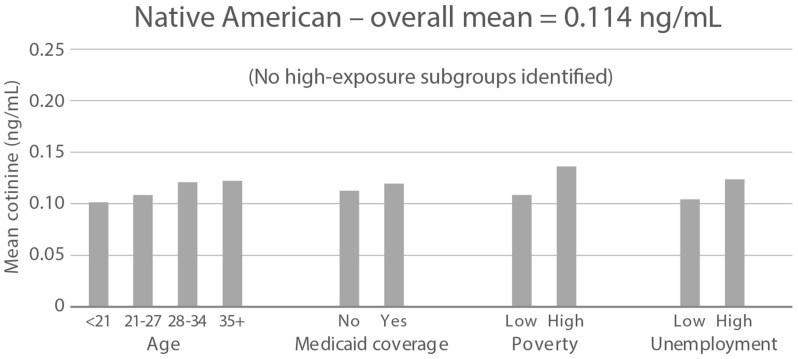
Geometric mean cotinine (ng/mL) in nonsmoker age and socioeconomic subgroups: Black and Native American high-ETS-level race/ethnicity groups. Highlighted bars indicate high-exposure subgroups.

High-exposure tobacco subgroups. Within the race/ethnicity groups with the highest tobacco exposure, subgroup smoking rates (Black, Native American, and White subjects), ETS rates, and ETS levels (Black and Native American subjects) are presented in Figure 2, Figure 3 and Figure 4. High smoking rates were identified in age groups <21 and 28–34 years and those having Medicaid coverage among Black subjects. High smoking rates were observed in ages 28–34 years and in those living in high-poverty census tracts among Native Americans, and in age group 21–27 and similarly those living in high-poverty census tracts among White subjects (Figure 2). Among Black subjects, age groups <21 and 21–27 and living in census tracts with high poverty were high-ETS subgroups, whereas among Native American subjects, age group 28–34 and having Medicaid coverage were high-ETS subgroups (Figure 3). High ETS levels were found in age group 21–27 and in those with Medicaid coverage among Black subjects; however, no high ETS level subgroups were identified among Native American subjects despite the high overall group ETS level (Figure 4).

**Figure 5:**
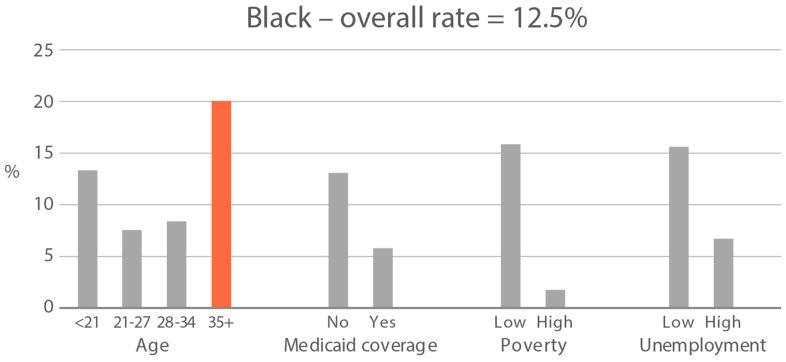

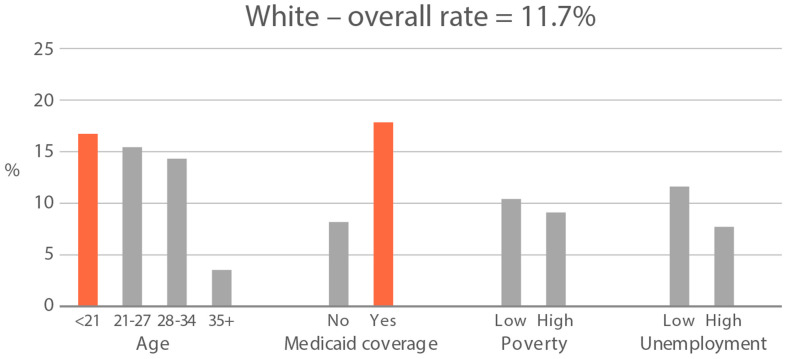
Percent of subjects with quantifiable OH-THC in age and socioeconomic subgroups: Black and White high cannabis exposure race/ethnicity groups. Highlighted bars indicate high-exposure subgroups.

**Figure 6:**
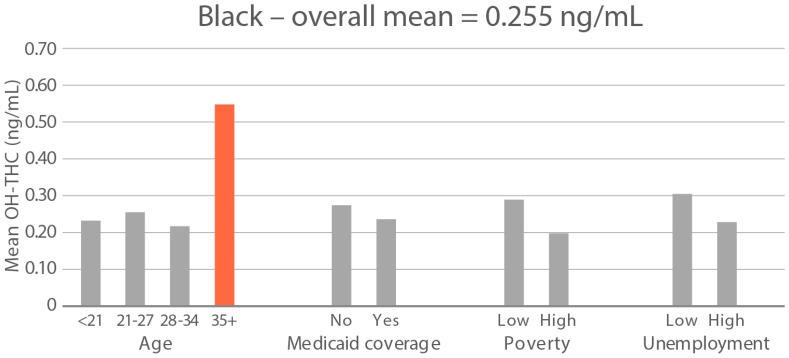

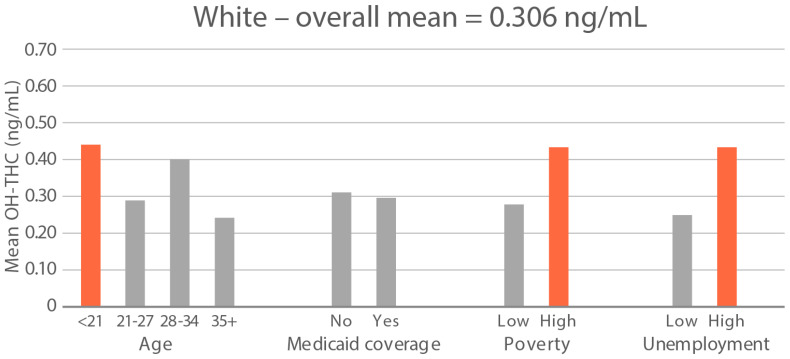
Geometric mean OH-THC (ng/mL) in age and socioeconomic subgroups: Black and White high-cannabis-level race/ethnicity groups. Highlighted bars indicate high-exposure subgroups.

High-exposure cannabis subgroups. Within groups with the highest cannabis detection and level (Black and White), subgroup detection rates and cannabis levels are presented in Figure 5 and Figure 6. The only Black subgroup with a high cannabis exposure rate and level was age 35+, although three higher-SES indicators consistently showed elevated rates and levels of cannabis below the high-exposure threshold. In the White group, high cannabis exposure rate subgroups were age <21 years and with Medicaid coverage. High cannabis level subgroups included age <21 and living in census tracts with high poverty and unemployment. Black and White groups showed contrasting high-cannabis-exposure subgroup profiles with older age and higher SES in the Black group, and younger age and lower SES in the White group.

**Figure 7:**
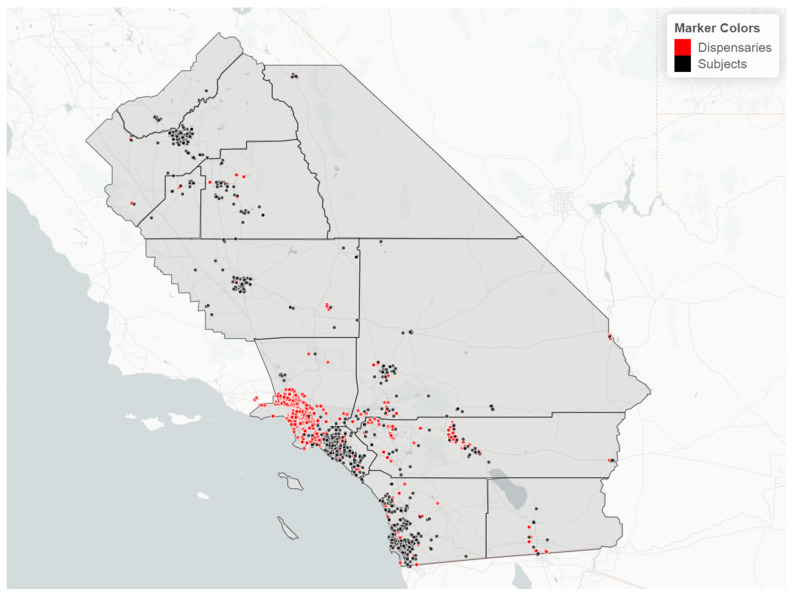
Map of study region with location of study subjects (black dots) and cannabis retailers (red dots), sample of 925 California prenatal screening program enrollees, 2018–2020.

**Figure 8:**
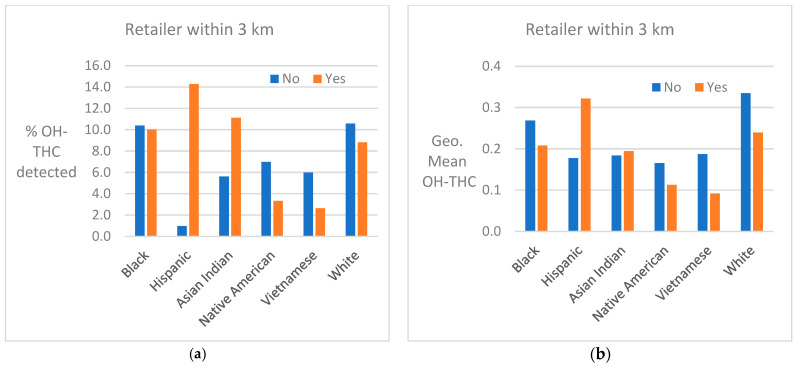
(**a**) Percent of race/ethnicity group with OH-THC detected by proximity to cannabis retailer, sample of California prenatal screening program enrollees, 2018–2020. (**b**) Geometric mean of OH-THC of race/ethnicity group by proximity to cannabis retailer, sample of California prenatal screening program enrollees, 2018–2020.

**Figure 9:**
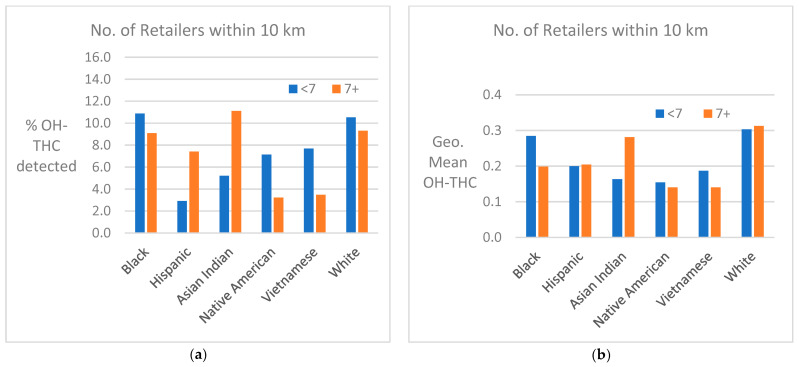
(**a**) Percent of race/ethnicity group with OH-THC detected by density of cannabis retailers, sample of California prenatal screening program enrollees, 2018–2020. (**b**) Geometric mean of OH-THC of race/ethnicity group by density of cannabis retailers, sample of California prenatal screening program enrollees, 2018–2020.

Cannabis retailer proximity and density. The location of study subjects and cannabis retailers is shown in Figure 7. In two race/ethnicity groups—Hispanic and Asian Indian—OH-THC rates were higher for subjects living in closer proximity to a cannabis retailer (within 3 km vs. more than 3 km) and living in an area with a greater density of cannabis retailers (seven or more retailers within 10 km vs. less than seven) (Figure 8a and Figure 9a). The Hispanic group living in an area with greater cannabis retailer density also had higher levels of cannabis than those living in an area with less density (Figure 9b), and the Asian Indian group had higher levels of cannabis if they lived in closer proximity to cannabis retailers (Figure 8b). Other race/ethnicity groups did not show increased cannabis levels with cannabis retailer proximity and density (Figure 8a,b and Figure 9a,b).

## 4. Discussion

We quantified prenatal exposure to tobacco and cannabis across six race/ethnicity study groups and identified exposure differences between groups and subgroups. When values of the assay below the detection limit were defined as unexposed, approximately 45–70% of subjects in each race/ethnicity group were exposed to tobacco while 5–13% were exposed to cannabis. The percent and level of ETS exposure in a race/ethnicity group tracked closely with the percentage of smokers in that group.

In our study population, tobacco and cannabis exposure did not always track together across race/ethnicity groups. Black, Native American, and White subjects were most highly exposed to tobacco and cannabis. Compared to the other groups, Black subjects had the highest rates of tobacco smoking, ETS exposure, and cannabis exposure; the highest level of ETS exposure and the second highest level of cannabis exposure. The Native American group had the second highest rate of tobacco smoking and level of ETS, but one of the lowest rates of cannabis exposure, as well as one of the lowest levels of cannabis. While the White group had the third highest rate of tobacco smoking and the fifth highest rates of ETS exposure, they had the second highest rate of cannabis exposure and the highest cannabis level. The Asian Indian group had the third highest ETS rates and one of the lowest ETS levels and fell into the middle ranges of rate and level of cannabis exposure. The Hispanic group was fourth highest in both rate and level of tobacco exposure, lowest in rate, and third highest in level of cannabis exposure. The Vietnamese group had the lowest rate and level of exposure to tobacco and the second lowest rate and level to cannabis. Knowledge of these differences in exposures may be useful in the selection, targeting, and specificity of public health interventions for race/ethnicity groups.

In a previous study (Hoshiko et al. 2019) [41], we ascertained rates of smoking and ETS exposure level in a population of prenatal screening program enrollees in a portion of southern California in 1999–2002 among 13 different race/ethnicity groups, including the six studied here. In this similarly designed study, we also analyzed serum cotinine as a marker for tobacco exposure. While it is not possible to compare rates and levels of ETS exposure because the two studies had assays with different cotinine sensitivities, it is possible to directly compare rates of smoking and rankings of ETS among the six race groups across the two studies. Compared to our previous study (Hoshiko et al. 2019) [41], the Black smoking rate increased from 12.7% in 1999–2002 to 16.1% in 2018–2020, and the ETS ranking remained the highest over these two time periods. In the Native American group, the smoking rate decreased from 12.7% to 9.6% and the ETS ranking increased from third to second highest over this time period. Tobacco smoking by White subjects increased slightly from 6.6% to 7.5% in 2018–2020, while the ETS ranking increased from the lowest to the third highest. The smoking rate in Hispanic subjects decreased from 3.1% to 2.0% and the ETS ranking increased from fifth to fourth highest. In Asian Indians, the smoking rate remained very low, increasing from 0.3% to 0.6%, and the ETS ranking changed from fourth to fifth highest. The Vietnamese group had similar smoking rates (0.7% and 0.6%) across the two studies, but showed a large decrease in ETS exposure, falling from the second highest to the lowest ranking over time. These findings suggest that there has been little progress in prenatal tobacco exposure reduction over the span of two decades in this region of California, especially in the highest exposed race/ethnicity groups.

Using 2017–2019 data from California’s Maternal and Infant Health Assessment (MIHA), an annual population-based survey of California resident women with a live birth, Azenkot et al. [44] found that cannabis use was more than twice as common as cigarette smoking among pregnant individuals (4.9% vs. 2.1%). These results were similar to those in our study in all race/ethnicity groups, except for Black and Native American subjects. Data from MIHA specific to the regions of California corresponding to the present study during 2018–2020 (n = 6900) revealed the highest pre-pregnancy smoking rate among Black (12.8%) and White (12.5%) participants, and a lower smoking rate among Asian/Pacific Islander (4.1%) and Hispanic (5.2%) participants. Smoking in the third trimester was rare (2.2% prevalence overall) and followed a similar pattern across race–ethnicity. MIHA data (limited to pre-pregnancy and the third trimester periods) do not reveal the two-fold Black–White disparity in smoking rates observed in our study using a second trimester biomarker. Cannabis use reported in MIHA for Black (11.9%) and Hispanic (4.7%) participants mirrors biomarker-based cannabis detection in our study; however, reported cannabis use during pregnancy among White subjects was much lower in MIHA (5.2%) than that detected in our study (11.7%). From 2018 to 2020, the overall rate of pre-pregnancy smoking in MIHA decreased (8.7% in 2018 vs. 6.8% in 2020) particularly among Black subjects (17.0% in 2018 vs. 9.8% in 2020), whereas overall cannabis use during pregnancy slightly increased (4.8% in 2018 vs. 5.5% in 2020), largely driven by an increase among White participants (4.3% in 2018 vs. 7.0% in 2020) [45]. There were no clear time trends identified in our six race/ethnicity groups from 2018 to 2020.

Young-Wolff et al. [46] examined cannabis use at ~8 weeks of gestation among a 2018 pregnant population served by Kaiser Permanente in 35 northern California counties. Based on self-report and toxicological urine testing, cannabis use was found in 26.6% of Black subjects, 8.8% in Hispanic subjects, and 8.6% in White subjects. Compared to rates in our study, the Kaiser results revealed rates twice as high for Black (12.5%) and Hispanic (4.5%) subjects and somewhat lower rates for White subjects (11.7%). A likely higher socioeconomic status of the Kaiser Permanente Northern California patient population [47] compared to our non-Kaiser southern California study population, especially in Black and Hispanic race/ethnicity groups, may account for these differences, as per the subgroup findings in our study. Another explanation could be that the Kaiser study focused on exposure in the first trimester while our study used blood specimens collected in the second trimester. Reported use of cannabis is higher in the first trimester than in the second trimester [48].

In this study, we have grouped together active users of cannabis and those passively exposed. There is some evidence in the literature that secondhand cannabis exposure may have contributed to the group disparities seen in this study. One study on prevalence of secondhand cannabis exposure found that about 27% of US adults reported past-week exposure to indoor and/or outdoor secondhand cannabis smoke, with similar exposure by sex and education [49]. Compared to White people, a higher percentage of Hispanic people and people of other non-Hispanic race/ethnicities reported past-week outdoor-only cannabis exposure, and a higher percentage of Black and Hispanic people reported both indoor and outdoor cannabis exposure.

With the objective of more finely targeting public health efforts to reduce exposure to both tobacco and cannabis, we examined subgroups within race/ethnicity groups that might be associated with greater exposure than the group as a whole. With regard to tobacco, exposure was greatest in younger subjects and those with indices of a lower economic status (i.e., Medicaid coverage, residence in census tracts with high poverty and unemployment) in Black, Native American, and White groups. With regard to cannabis exposure, similar subgroups were associated with high exposure in Native American and White race/ethnicity groups. In contrast, Black subjects in older age categories and those with at least one of three indices of a higher economic status were found to be positively associated with exposure. The contrasting subgroup profiles for exposure to tobacco and cannabis in Black subjects have not been observed before and warrant further exploration. In studies previously conducted, most prior to cannabis legalization, characteristics of pregnant individuals who use cannabis are similar to those who are tobacco-exposed, e.g., less education, higher unemployment, and lower household income, compared with nonusers [50,51]. As cannabis exposure increases and tobacco smoking decreases over time, a different high-cannabis characteristic user profile may emerge. This proposition is supported by a northern California study that found increasing use of cannabis without other substances between 2009 and 2018, and decreased co-use of cannabis with other substances like nicotine, pharmaceutical opioids, and stimulants [52]. Laws legalizing cannabis can further impact cannabis use and may contribute to contrasting profiles post-legalization. For example, the age of cannabis users is higher in states with laws allowing medical or recreational adult use than in states without such laws [53].

Proximity or density of cannabis retailers was positively associated with detection of cannabis in two race/ethnicity groups (Hispanic and Asian Indian). A similar positive association was found with cannabis retailers and cannabis use in a study of Kaiser Permanente northern California prenatal patients—53% of whom were Hispanic or Asian/Pacific Islanders—in 2018, the first year that recreational cannabis was legalized [44]. It is not clear whether there were underlying differences in this association by race/ethnicity, as we found in our study. In a systematic literature review of the impact of legal cannabis availability on cannabis use, authors concluded that groups who have been least exposed to cannabis before legalization may be most susceptible to increased availability [54].

In our study, exposure to tobacco was positively related to cannabis in most but not all race/ethnicity groups. In a retrospective cohort study of prenatal clinic attendees in Baltimore in 2009–2010, marijuana use was strongly correlated with cigarette use [51].

This study has many strengths due to the use of population-based, stored specimens. The study population we used was unlikely to be impacted by selection bias because the study sample was randomly chosen from the population of California prenatal screening enrollees. The study sample was selected retrospectively from a base population of approximately 190,000 prenatal screening participants in a manner to include three groups not usually studied separately (Native American, Asian Indian, and Vietnamese), providing more specificity in understanding patterns of use in California than previous investigations could. The assay developed for this study is unique and had the capability to analyze tobacco and cannabis biomarkers simultaneously with a high throughput, although only one of the three cannabis biomarkers was reportable. The use of serum biomarkers provided an objective measurement of the two exposures. The five methods of evaluating tobacco and cannabis exposure permitted differentiation between the exposure rate (focused on the percentage of subjects with the specific, usually highest, exposure) and level (the mean concentration) of the exposure. The exposure rate and exposure level may not always track together. For example, compared to other groups, the Vietnamese group had a higher rate of ETS-exposed subjects but a lower level of ETS exposure, and the White group had a lower rate of ETS exposure but a higher level of ETS exposure. Knowledge of exposure rate and level can help in developing specific exposure prevention approaches, recommendations, and messaging.

While the study has many strengths, there are several limitations as well. The biomarkers were measured at a single point in time, which may or may not reflect a subject’s exposure at other times during pregnancy. As such, the rates of exposure we reported may underestimate whole-pregnancy exposure. The cannabis biomarker (OH-THC) reflects exposure only to THC and not to other compounds in cannabis that people use, like CBD. Unlike serum cotinine, which has an approximate half-life of 16 h [55] and reflects a consistent exposure in the population to nicotine in tobacco, interpreting a single serum value of OH-THC poses a challenge. The half-life of this THC metabolite (OH-THC) in serum is 12–36 h [56] and unlike cotinine, there are no predefined cutoff points to distinguish active use of cannabis from environmental exposure. With a newly developed and extensive laboratory method, analytical challenges arose, especially in the reliability of quantitating the additional desired cannabis exposure biomarkers, CBN and Δ9-THC. Thus, data for these compounds could not be reported. While detection limits for cotinine and OH-THC were not as low as other assays [57,58], the use of analytical instrument values below these limits allowed us to examine the full distribution of the analytes. The reduced sample size increased the variability of our estimates and precluded statistical comparisons. Proximity and density of cannabis retailers represents only one of many ways in which pregnant individuals access cannabis. Other sources include the black market, online retailers, or via personal networks not located in their residential area. Such factors could differ by race/ethnicity groups and impact the cannabis retailer findings we observed.

## 5. Conclusions

Compared to the other race/ethnicity groups studied, Black and White subjects had the highest rates and levels of cannabis exposure in the study population of prenatal screening enrollees in the first three years after cannabis legalization in California. During this time, Black and Native American subjects had the highest rates of tobacco smoking and rates and levels of ETS exposure. Knowledge of race/ethnicity, in addition to factors such as age, Medicaid insurance coverage, and residence in high-poverty neighborhoods, can help identify communities at a higher risk of these exposures. In most groups, exposure to cannabis was positively associated with exposure to ETS, and even more strongly associated with tobacco smoking. All of these factors should be considered in targeting populations for public health interventions aimed at cannabis and tobacco exposure reduction in pregnancy. Such actions are necessary to ensure that existing health inequities are not further exacerbated.

## Figures and Tables

**Table 1:** Percent of subjects in six race/ethnicity study groups for selected individual and neighborhood characteristics, sample of California prenatal screening program enrollees, 2018–2020.

	Race/Ethnicity Group					
Characteristic (Total N = 925)	Black (*n* = 161)	Hispanic (*n* = 147)	Asian Indian (*n* = 156)	Native American (*n* = 136)	Vietnamese (*n* = 164)	White (*n* = 161)
Age at blood collection (years) ^1^						
<21	10.6	10.2	0.0	12.5	1.2	4.4
21–27	38.5	34.0	9.6	33.1	7.9	19.3
28–34	34.8	34.7	50.6	36.0	45.1	36.7
≥35	16.2	21.1	39.7	18.4	45.7	39.8
Medicaid insurance coverage (%) ^1^	34.8	43.5	16.0	20.6	16.5	19.3
County of residence (%) ^1,2^						
Fresno	18.0	14.3	18.6	5.9	1.8	5.0
Imperial	1.2	4.8	0.0	0.0	0.0	0.0
Inyo	0.0	0.0	0.0	4.4	0.0	0.0
Kern	10.6	7.5	14.1	11.1	0.0	3.1
Kings	0.6	2.0	0.6	3.0	0.0	3.1
Los Angeles	1.9	3.4	1.3	2.2	3.7	1.9
Madera	0.0	4.1	0.6	1.5	0.0	0.0
Merced	0.0	0.0	0.6	0.0	0.0	0.0
Orange	6.2	12.9	28.9	17.0	70.7	29.2
Riverside	5.0	6.8	0.0	6.7	3.1	6.2
San Bernardino	21.7	4.1	1.9	11.1	0.0	8.1
San Diego	32.9	27.9	33.3	30.4	20.7	38.5
Tulare	1.9	12.2	0.0	6.7	0.0	5.0
≥20% below poverty level (%) ^1^	45.3	34.0	8.3	29.4	12.2	16.2
≥6% unemployed (%) ^1^	67.7	64.0	34.0	54.4	32.9	39.8
Cannabis retailer ≤ 3 km from residence (%) ^2,3^	22.7	22.1	21.1	24.4	21.7	27.5
Number of cannabis retailers within ≤10 km radius (mean) ^1,2^	6.4	6.0	5.7	5.8	12.6	7.2

^1^ Chi-square or ANOVA *p*-value < 0.0001. ^2^ Percentages reflect one person excluded from these analyses based on geographic residence outside study area. ^3^ Chi-square *p*-value = 0.774.

**Table 2:** Tobacco (a) and cannabis (b) exposure singularly and jointly (c) according to cotinine and OH-THC biomarkers by race/ethnicity study groups, sample of California prenatal screening program enrollees, 2018–2020.

	**Race/Ethnicity Group**					
	**Black**	**Hispanic**	**Asian Indian**	**Native American**	**Vietnamese**	**White**
Tobacco						
Categorical exposure						
No. of subjects (total N = 925)	161	149	158	136	165	163
Cotinine ≥ 0.1 ng/mL (%, 95% CI)	69.6 (62.4, 76.4)	48.3 (40.1, 56.5)	50.6 (42.7, 58.6)	60.3 (52.0, 68.6)	44.5 (36.8, 52.2)	53.4 (45.6, 61.2)
Smoker ^1^ (%, 95% CI)	16.1 (10.4, 21.9)	2.0 (0.0, 4.4)	1.3 (0.0, 3.1)	9.6 (4.6, 14.6)	0.6 (0.0, 1.8)	7.5 (3.4, 11.6)
ETS, nonsmokers with cotinine ≥0.1 ng/mL ^2^ (%, 95% CI)	53.4 (45.6, 61.2)	46.3 (38.1, 54.4)	49.4 (41.4, 57.3)	50.7 (42.2, 59.2)	43.9 (36.2, 51.6)	46.0 (38.2, 53.7)
Continuous exposure						
No. of nonsmoking subjects (total N = 826)	128	139	147	113	157	142
Cotinine, ng/mL (geometric mean, 95% CI) ^3^	0.153 (0.121, 0.194)	0.090 (0.080, 0.103)	0.085 (0.074, 0.097)	0.114 (0.092, 0.138)	0.085 (0.075, 0.097)	0.093 (0.078, 0.111)
Cotinine, ng/mL (IQR 25–75th percentile)	0.075–0.240	0.064–0.140	0.060–0.146	0.071–0.183	0.054–0.138	0.056–0.157
	**Race/Ethnicity Group**					
	**Black**	**Hispanic**	**Asian Indian**	**Native American**	**Vietnamese**	**White**
b. Cannabis						
Categorical exposure						
No. of subjects (total N = 677)	112	112	117	98	118	120
OH-THC ≥ 1.0 ng/mL (%, 95% CI)	12.5 (6.3, 18.7)	4.5 (0.6, 8.3)	7.7 (2.8, 12.6)	7.1 (2.0, 12.3)	5.9 (1.6, 10.3)	11.7 (5.8, 17.5)
Continuous exposure						
No. of subjects with OH-THC values >0 ng/mL (total = 397)	76	58	76	51	62	74
OH-THC, ng/mL (geometric mean, 95% CI) ^3^	0.255 (0.186, 0.350)	0.201 (0.146, 0.277)	0.186 (0.131, 0.264)	0.150 (0.088, 0.256)	0.156 (0.105, 0.233)	0.306 (0.223, 0.419)
OH-THC, ng/mL (IQR 25–75th percentile)	0.100–0.700	0.070–0.480	0.090–0.480	0.050–0.510	0.707–0.470	0.170–0.750
	**Race/Ethnicity Group**					
	**Black**	**Hispanic**	**Asian Indian**	**Native American**	**Vietnamese**	**White**
c. Cannabis and Tobacco						
Categorical exposures						
No. of subjects (total N = 677)	112	112	117	98	118	120
OH-THC (≥1 ng/mL): -In smokers ^1^ (%, 95% CI)	31.8 (10.7, 53.0)	N/A	N/A	8.3 (0.0, 26.7)	N/A	50.0 (5.3, 94.7)
-In ETS-exposed nonsmokers ^2^ (%, 95% CI)	8.5 (1.2, 15.8)	7.1 (0.2, 14.1)	7.7 (0.2, 15.2)	9.1 (1.2, 16.9)	8.2 (0.2, 16.1)	13.7 (4.0, 23.5)
-In nondetects, cotinine < 0.1 ng/mL (%, 95% CI)	6.5 (0.0, 15.6)	1.9 (0.0, 5.7)	6.3 (0.2, 12.5)	3.2 (0.0, 9.8)	4.3 (0.0, 9.3)	4.9 (0.0, 10.5)
Cotinine (≥0.1 ng/mL):						
-In OH-THC-detected subjects (%, 95% CI)	85.7 (64.7, 100)	80.0 (24.5, 100)	55.6 (15.0, 96.1)	85.7 (50.8, 100)	57.1 (7.7, 100)	78.6 (54.0, 100)
-In nondetects, OH-THC < 1 ng/mL (%, 95% CI)	70.4 (61.2, 79.6)	51.4 (41.8, 61.0)	45.4 (35.8, 54.9)	67.0 (57.2, 76.9)	40.5 (31.3, 49.8)	45.3 (35.7, 54.9)
Continuous exposures						
No. of subjects (total N = 384) ^3^	72	56	74	51	60	71
Pearson correlation coefficient, cotinine and OH-THC (*p*-value)	0.25 (0.03)	−0.10 (0.45)	−0.05 (0.64)	−0.04 (0.78)	0.33 (0.01)	0.42 (0.00)

^1^ As per Benowitz et al. (2008) [43]; ^2^ ETS in nonsmokers had serum cotinine values ≥0.1 ng/mL and below smoker cutoff points; ^3^ Analysis includes subjects with values (>0 ng/mL).

## Data Availability

The data presented in this study are available on request from the corresponding author and approval from the California Biobank Program. The data are not publicly available due to privacy restrictions of the California Prenatal Screening Program.

## Supplementary Materials

The following supporting information can be downloaded at: https://www.mdpi.com/article/10.3390/ijerph21010011/s1, Figure S1: Distribution of log_10_ cotinine for nonsmokers by study group (zero values set to leftmost bar), sample of California prenatal screening program enrollees, 2018–2020; Figure S2: Distribution of log_10_ OH-THC by study group (zero values set to leftmost bar), sample of California prenatal screening program enrollees, 2018–2020.
