# Supplementary material for: Prenatal Exposure to Tobacco and Cannabis in Six Race/Ethnicity Groups during the First Three Years after Legalization of Cannabis for Recreational Use in California
Source: Int J Environ Res Public Health. 2023 Dec 21;21(1):11. doi: 10.3390/ijerph21010011 (PMC10815235; doi:10.3390/ijerph21010011)
Supplement: Supplementary file 1 [file ijerph-21-00011-s001.zip › ijerph-2746813-supplementary.pdf]

Supplemental Materials

Figure S1. Distribution of $\log_{10}$ cotinine for nonsmokers by study group (zero values set to leftmost bar), sample of California prenatal screening program enrollees, 2018-2020.

Black Hispanic Asian Indian

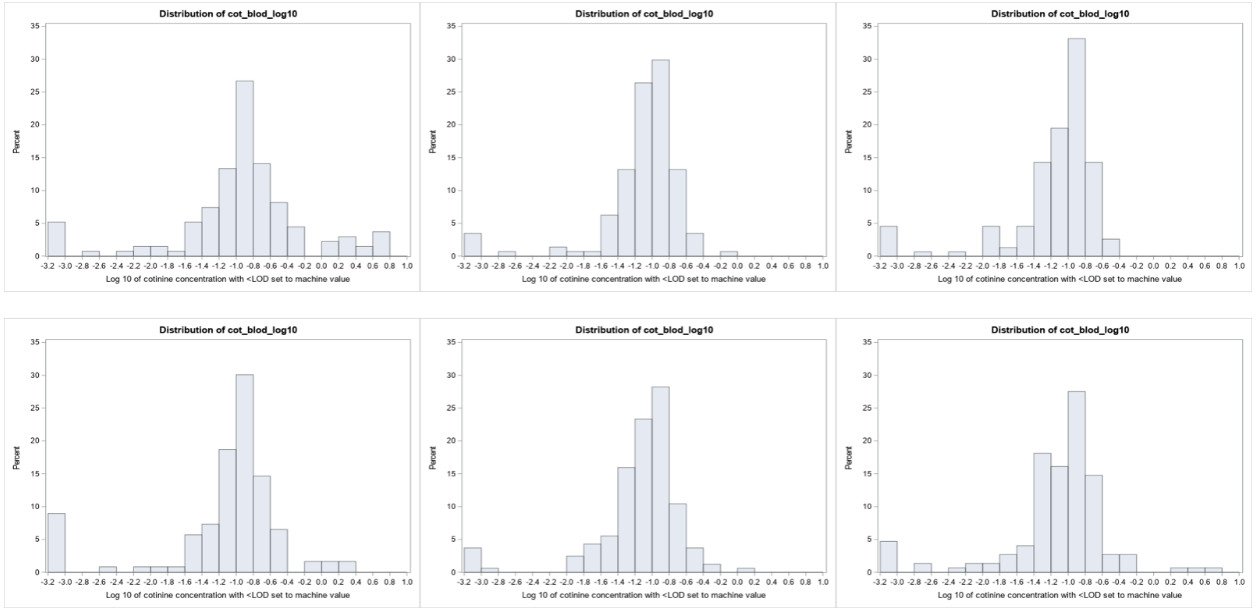

Native American Vietnamese White

Figure S2. Distribution of $\log_{10}$ OH-THC by study group (zero values set to leftmost bar), sample of California prenatal screening program enrollees, 2018-2020.

Black Hispanic Asian Indian

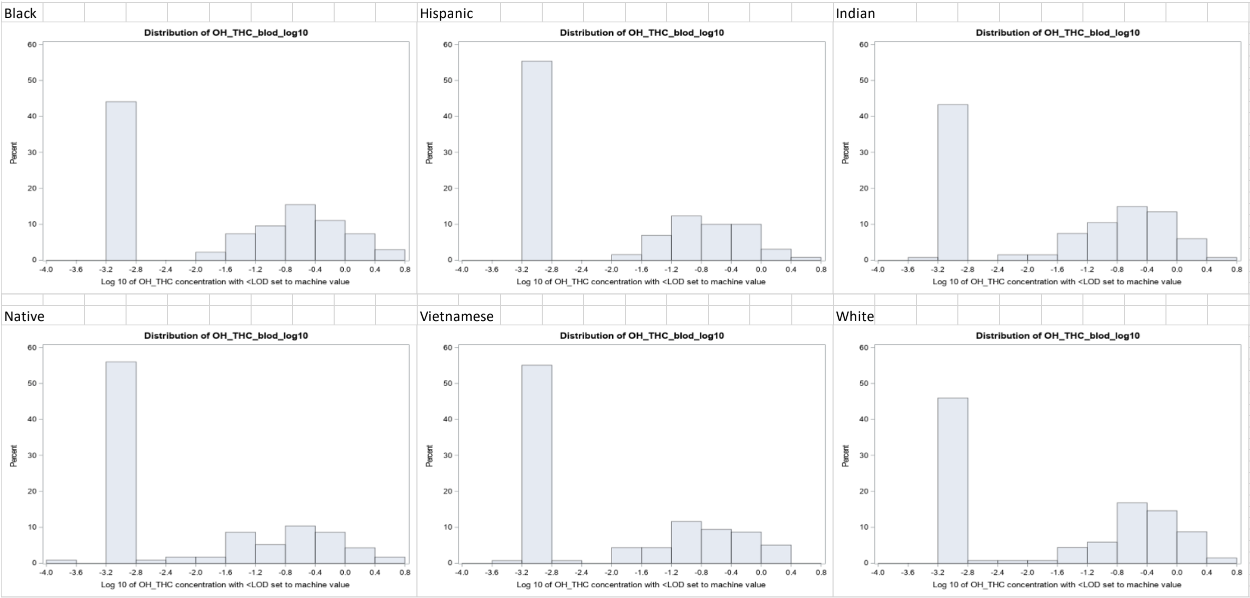

Native American Vietnamese White
